# Exploring the relationships between ground observations and remotely sensed hazelnut spring phenology

**DOI:** 10.1007/s00484-024-02815-1

**Published:** 2024-11-08

**Authors:** Sofia Bajocco, Mara Di Giulio, Abdoul Hamid Mohamed Sallah, Simone Bregaglio

**Affiliations:** 1 Research Centre for Agriculture and Environment (CREA-AA), CREA-Council for Agricultural Research and Economics, Via della Navicella 2-4, Rome, 00184 Italy; 2 Ferrero Hazelnut Company, Ferrero Trading Lux, Senningerberg, Luxembourg; 3 Council for Agricultural Research and Economics, Research Centre for Agriculture and Environment (CREA-AA), Via di Corticella 133, Bologna, 40128 Italy

**Keywords:** MODIS, Spring phenology, Hazelnut, EVI, Growing season

## Abstract

Crop phenology is very important in regular crop monitoring. Generally, phenology is monitored through field observation surveys or satellite data. The relationships between ground observations and remotely sensed derived phenological data can enable near-real-time monitoring over large areas, which has never been attempted on hazelnuts. In this study, we extracted phenological metrics derived from MODIS Enhanced Vegetation Index (EVI) in hazelnut production regions and compared them with the spring ground phenological data (BBCH scale) from orchards located in the same area of Turkey over the period from 2019 to 2022. We observed a specific temporal dynamic between remote sensing phenometrics and ground observations. The metrics Greenup, Upturning Date, and Threshold 20% metrics corresponded to the early of EVI growth and were synchronous with the female flowering of hazelnut and ending before bud break. The metrics Threshold 50% and Start of season were associated with the steepest portion of the EVI curve, i.e., canopy greening and thickening, and occurred between ovaries enlargement and leaves unfolding. The metrics Peak of Season, Stabilization Date, and Maturity corresponded to the end of spring vegetative growth. The main outcomes are that (i) female flowering occurred before 20% of vegetation development (BBCH 64P occurred about one month before Threshold 20%), (ii) phenometrics from satellite remote sensing (i.e., Upturning Date and Threshold 20%) well-reflected leaf emergence (r_s_ = 0.30 and r_s_ = 0.32, respectively; *p* < 0.05) and unfolding (r_s_ = 0.35 and r_s_ = 0.39, respectively; *p* < 0.05), and (iii) cluster appearance temporally aligned with the peak of the EVI curve (Stabilization Date and BBCH 71P differed by around 4 days). Our method is transferable to operational phenology monitoring, and future applications will consider the senescence season and the effect of environmental variability on the comprehension of vegetation dynamics.

**Supplementary Information:**

The online version contains supplementary material available at 10.1007/s00484-024-02815-1.

## Introduction

European hazelnut (*Corylus avellana* L.) is a deciduous tree from the Birch family, naturally distributed in temperate Mediterranean regions (FAO 2018). It ranks as the fifth most popular nuts tree globally, with 528,068 Mg of kernels produced in 2019, following almonds, walnuts, cashews, and pistachios (International Nut and Dried Fruits Council 2019). Despite hazelnuts’ broad natural distribution, commercial orchards are concentrated in a few countries, with Turkey accounting for nearly 65% of global production (Islam 2018), followed by Italy, USA, and Azerbaijan (FAO 2018). Given the prominence and heterogeneity of the Turkish hazelnut growing areas, the comprehension of hazelnut phenological dynamics becomes valuable for explaining the interannual yield variability and enabling in-season vegetation monitoring (Paradinas et al. 2022).

Phenology is the study of recurring biological events in relation to biotic and abiotic factors, and the relationships between phases of the same and different species (Lieth 1974). This field of study encompassing biometeorology, ecology, and evolutionary biology (Morellato et al. 2016), which has gained significance in global change research. Phenology is crucial for understanding the growth dynamics of horticultural crops, including hazelnut, where it can aid crop management actions, yield prediction, frost risk alerts, and early advice for pest outbreaks and fungal infections (Valeriano et al. 2022; Bregaglio et al. 2021).

Researchers have studied plant phenology at multiple spatial and temporal resolutions and with various analytic tools (Cleland et al. 2007; Balzarolo et al. 2016). The conclusions regarding phenological transitions are influenced by how the initial measurements were combined across spatial, temporal, and taxonomic dimensions (Park et al. 2021). Two main approaches exist: small-scale, ground-based investigations (Workie and Debella 2018), and large-scale assessments utilizing satellite remote sensing (Broich et al. 2015; D’ Odorico et al. 2015). Ground observations classify phenological stages according to observational scales (Meier et al. 2009), and a new system based on the Biologische Bundesanstalt Bundessortenamt and Chemische Industrie (BBCH) scale has been recently proposed for hazelnut (Taghavi et al. 2022), synthesizing previous country- (French, Germain and Sarraquigne 2004; Italy, Malossini 1993) or stage-specific (flowering, Capik and Molnar 2014) methods. Nevertheless, the availability of homogenized datasets of hazelnut phenological data has been hampered by the time and efforts required to acquire field data and by the lack of specific research programs.

Remote sensing can overcome some of these limitations, bridging the gap between traditional phenological studies and the broader perspective provided by global models (Garonna et al. 2018). Remotely sensed phenology refers to the seasonal variation in spectral vegetation indices (such as greening, senescence, and dormancy) observed by satellites (Friedl et al. 2006; Bajocco et al. 2019a). However, satellite observations present a spatially aggregated signal derived from diverse surface conditions, which may not accurately represent the responses of specific plant species, as the pixel size varies from meters to kilometers. Available techniques rely on deriving vegetation indices (VIs), such as the normalized difference vegetation index (NDVI, Rouse et al. 1974) or the enhanced vegetation index (EVI, Huete et al. 2002), which are measures of vegetation “greenness”. These indices act as proxies for photosynthetic activity, utilizing the interaction of visible light with leaf pigments and near-infrared (NIR) energy with internal leaf and canopy structures (D’Odorico et al. 2015).

The temporal dynamic of satellite remotely sensed VIs aligns strongly with typical vegetation growth stages, therefore analyzing their curves enables quantifying intra-annual changes in the timing and intensity of vegetation activity, allowing the extraction of phenological metrics related to the growing season (i.e., phenometrics), such as the start, end, and duration (Reed et al. 1994; Ferrara et al. 2023). Various authors have derived phenometrics using different smoothing/fitting methods (e.g., Savitzky-Golay filter, harmonic analysis, logistic and double logistic) and extraction techniques (e.g., thresholds, derivatives, regression tree model) (for a review, see Zeng et al. 2020). Remote and proximal sensors have been recently used to study the architecture of the hazelnut plantations (Li 2021; Altieri et al. 2022; Vinci et al. 2023) and to identify hazelnut orchards in anthropogenic landscapes (Martelli et al. 2023). However, to our knowledge, hazelnut phenology has never been explored with remote sensing.

From this perspective, this study aims to explore hazelnut phenology at a regional scale by analyzing temporal and physiological relationships between satellite remote sensing phenometrics and phenological ground observations in Turkish orchards distributed across two hazelnut production regions. Specific objectives are: (i) to compute phenometrics from remote sensing using alternative fitting models, (ii) to analyze their correlation with the timing of ground-observed vegetative and reproductive phases.

## Materials and methods

### Study area

Turkey holds the title of being the birthplace of hazelnut cultivation and boasts the position of being the world’s largest producer and exporter of hazelnuts (Erdogan 2018). Approximately 700,000 hectares of Turkish land are dedicated to hazelnut cultivation (Turkish Statistical Institute 2020).

**Fig. 1:**
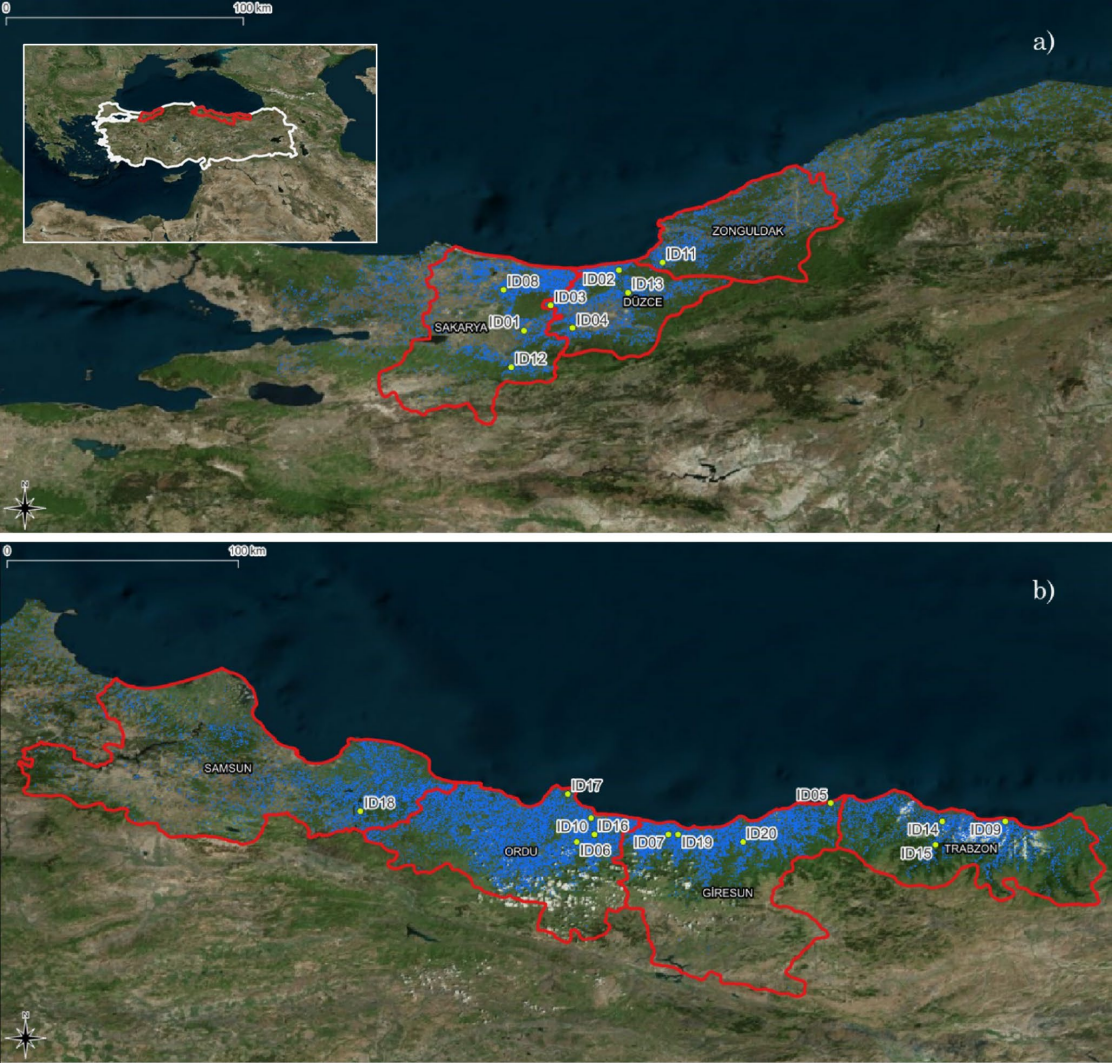
Geographical locations of the surveyed orchards (yellow dots) in the seven main Turkish-producing municipalities (red borders) placed in the West (**a**) and East (**b**) Black Sea area. In blue, the hazelnut distribution map of 2019. (source: Ferrero Hazelnut Company)

Traditionally, two distinct hazelnut production regions have been identified in Turkey, each with unique characteristics (Fig. 1): the Eastern region covering the Georgian border to the Central Black Sea coast and the Western region comprising the Central and Western Black Sea coasts. With its favorable climate, the Eastern region is considered the most suitable for hazelnut cultivation (Erdogan 2018). Orchards in this region are typically small (less than 2 hectares), predominantly old (over 50 years), and often lack mechanization. The landscape is characterized by narrow coastal plains and steep hills or mountains running parallel to the sea, resulting in hazelnut planting extending up to 30 km inland. In costrast,, the Western region features relatively flat or gently sloping land, facilitating mechanization. The hazelnut trees in this area grow in fertile soils, and the orchards are well-organized with larger average size and younger trees, contributing to higher yields compared to the Eastern region.

### Ground observations

**Fig. 2:**
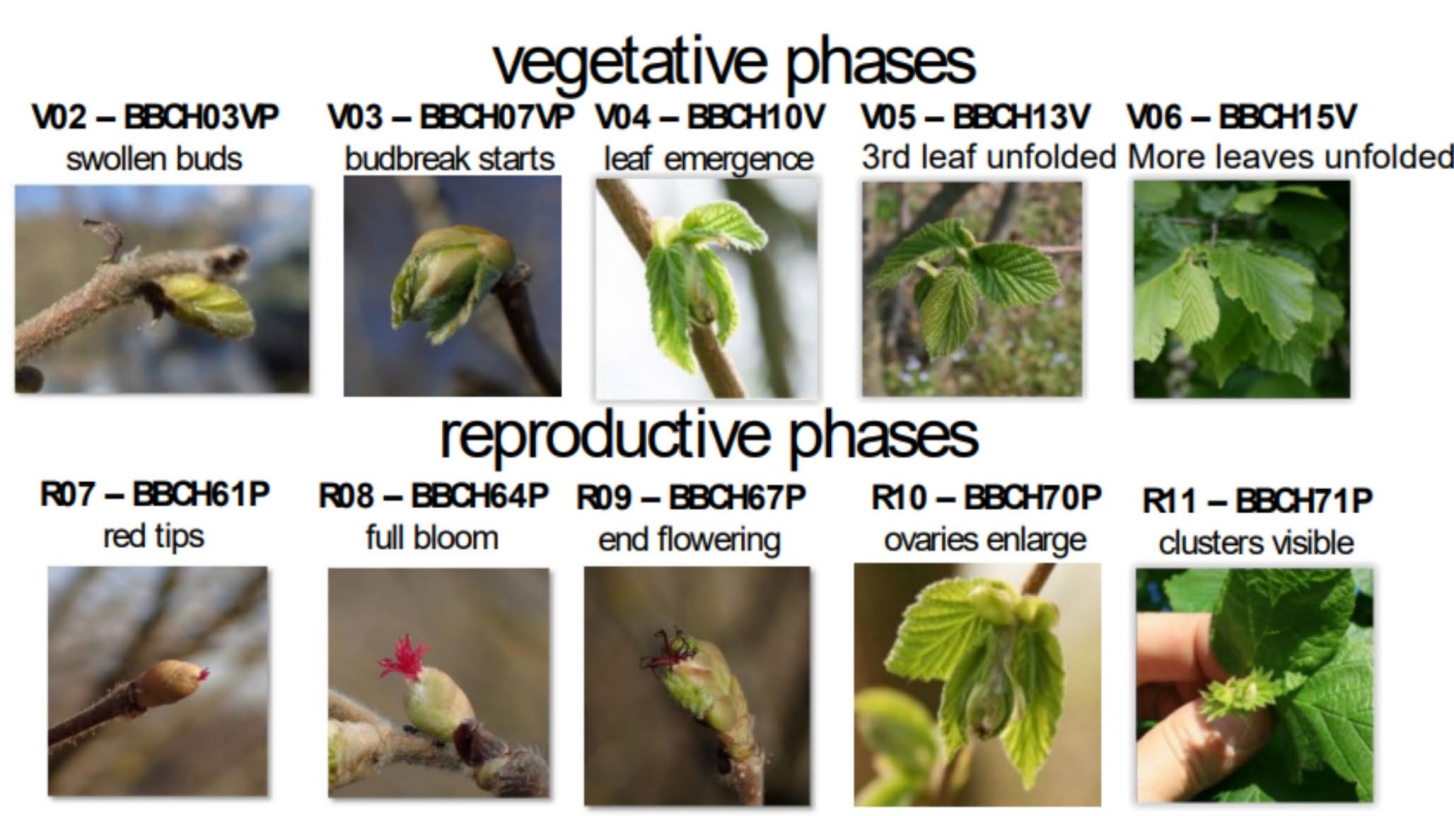
Visual representation and synthetic description of main hazelnut spring phenology phases and correspondent codification according to the Italian Phenological Garden system and Biologische Bundesanstalt Bundessortenamt and Chemische Industrie (BBCH) scales

Starting from October 2018, the Agri Competence Centre of Ferrero Hazelnut Company launched a phenological survey program on 20 hazelnut orchards placed in key Turkish producing areas (provinces Sakarya, Duzce, Zonguldak in the Western region; provinces Giresun, Ordu, Samsun, Trabzon in the Eastern region, Fig. 1). The prevalent planting spacing scheme among these orchards is 4 m × 4 m. Four primary hazelnut varieties are cultivated in these orchards: Çakildak, Mincane, Tombul, and Palaz. Three other hazelnut varieties, namely Sivri, Fosa, and Kara Findik are grown. The orchards’ altitudes range from 61 m to 1010 m a.s.l. The orchards are evenly distributed across this elevation range with seven orchards below 250 m a.s.l., nine orchards below 500 m a.s.l. and the remaining four situated at higher elevation. Since 2019, a team of two/three agronomists visit each orchard weekly to assess hazelnut phenology through an online Google form (Supplementary Material S1). Each agronomist independently assesses male and female reproductive stages, and vegetative stages on ten hazelnut trees by comparing visual observations with infographics and textual description of the phases, using an expert-modified version of the Italian Phenological Garden system scale (Malossini 1993). In total, 684 observations related to hazelnut spring phenology have been quality-checked and converted to the new 3-digit BBCH codes from Taghavi et al. (2022), according to their definition and description (Fig. 2). This code describes the stages of progression from leaf budding to stem elongation to leaf fall.

### Remote sensing observations

The time series of satellite imagery used to estimate hazelnut phenometrics were obtained using Google Earth Engine from MODIS (Moderate-resolution Imaging Spectroradiometer) MOD13Q1 and MYD13Q1 maximum value composite (MVC) Enhanced Vegetation Index (EVI) products. The data was collected from the MODIS Terra and Aqua sensors, covering the period from 2019 to 2022, and according to the hazelnut map of Turkey of 2019 (Fig. 1; source: Ferrero Hazelnut Company). The spatial resolution of the imagery was 250 m, and the temporal frequency was 8 days, resulting in a total of 46 images per year, from January 1st to December 27th. The MVC procedure is employed to reduce noise in the available time series for each pixel by selecting only the highest EVI value within a specific period as a representative (Holben 1986). This technique maintains a consistent temporal resolution while minimizing the influence of atmospheric and geometric effects in optical imagery. To optimize the vegetation signal further and mitigate the impact of canopy background and atmospheric effects, the EVI (-1; 1) was computed (Eq. 1, Huete et al. 2014; Broich et al. 2015):

1 $$\eqalign{& {\rm{EVI}}\,{\rm{ = }}\,{\rm{G \times }}\left( {{\rm{\rho NIR--\rho red}}} \right) \cr & {\rm{/}}\left( {{\rm{\rho NIR + }}{{\rm{C}}_{\rm{1}}}{\rm{ \times \rho red - }}{{\rm{C}}_{\rm{2}}}{\rm{ \times \rho blue + L}}} \right) \cr} $$

where $\:G$ is the gain factor, ρ is the surface reflectance (atmospherically corrected), $\:{C}_{1}\:$and$\:\:{C}_{2}$ are the coefficients of the aerosol resistance term, and $\:L$ is the canopy background adjustment. The coefficients used in the EVI algorithm were $\:L$ = 1, $\:{C}_{1}$ = 6, $\:{C}_{2}$= 7.5, and $\:G$ = 2.5 (Huete et al. 2002).

**Fig. 3:**
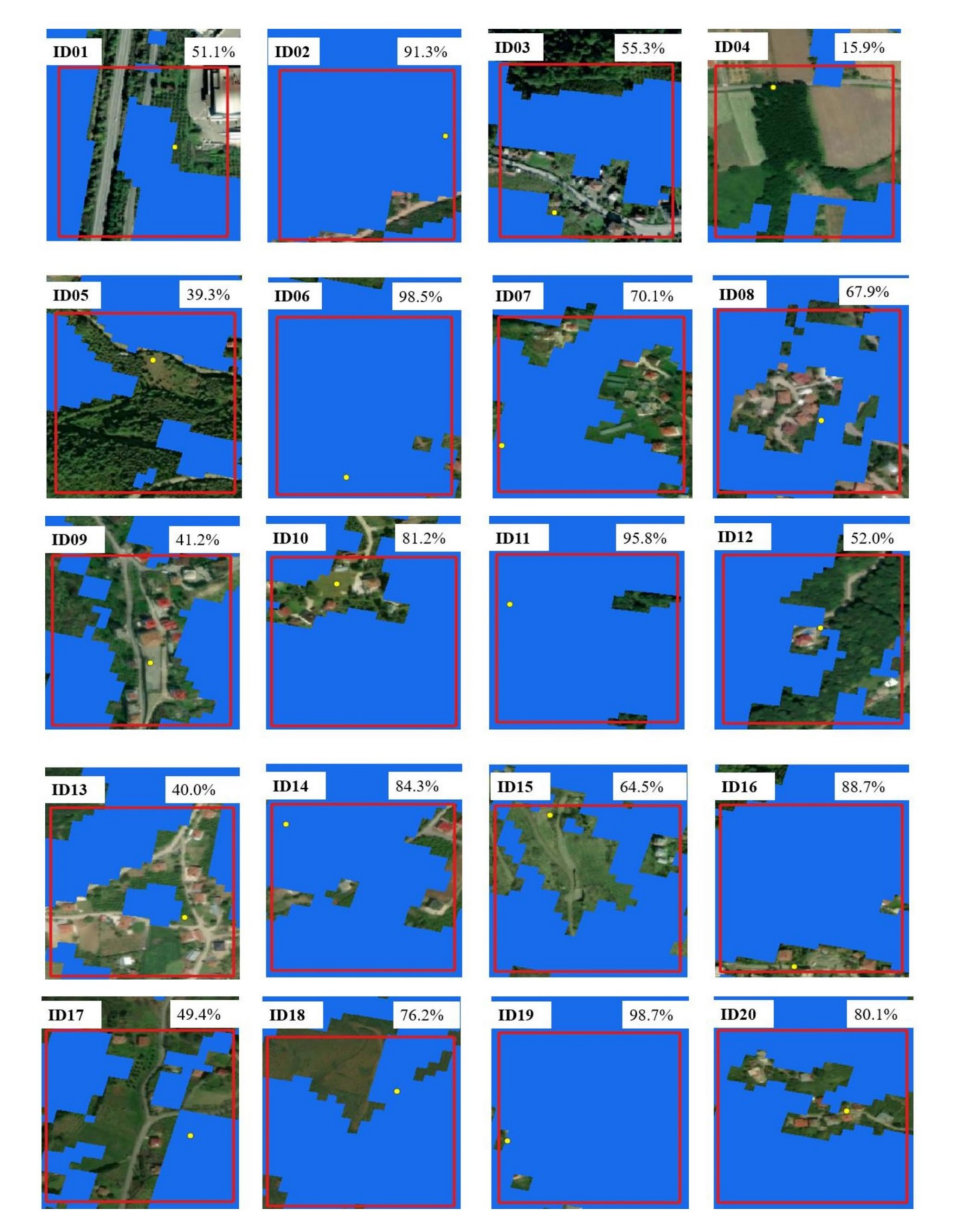
Percentages of hazelnut coverage in the MODIS pixels (red boxes) in which the 20 hazelnut orchards (yellow dots) fall. The hazelnut distribution map of 2019 is shown in blue

Although the MODIS coarse spatial resolution may introduce some uncertainties in accurately defining hazelnut phenological events, it is reasonable to expect that the hazelnut’s radiometric response is adequately represented since the landscape is predominantly occupied by extensive hazelnut cultivation. Specifically, in 15 out of the 20 MODIS pixels used, hazelnut cultivation covered more than 50% of the pixel area (Fig. 3). Finally, considering the landscape characteristics and our objective of studying hazelnut phenology at a regional scale rather than focusing on individual fields, the use of a sensor like MODIS is justified.

### Phenometrics extraction

Time series of MODIS EVI have been quality-checked and filtered according to expert-derived rules, i.e., EVI > 0.1 throughout the year and EVI > 0.3 in June-August. The total number of EVI data discarded from the analysis by the expert-based filter was 124 out of 3540 (3.5%). A third-degree polynomial Savitzky-Golay (SG) filter has been applied to smooth EVI temporal dynamics, with the parameters adjusted based on expert inspection of the curves (Chen et al. 2004). The half-width of the smoothing window has been set to 9 and the degree of the polynomial to 3. The SG filter allowed the cleaning of all the spurious EVI values related to atmospheric effects and signal noise while keeping the observed trend of canopy temporal dynamics. Daily EVI time series were computed with the Klos double logistic fine-fitting method as implemented in the *phenofit* R package (Kong et al. 2022). Spring phenological metrics were extracted from daily time series using alternative methods: (i) Threshold method, (ii) Derivative method, (iii) Gu method, and (iv) Zhang method (Table 1). In the threshold method, start of season (SOS) is defined as the day of the year when a vegetation index exceeds a given threshold, i.e., the 20th and 50th percent of the annual amplitude (TRS2 SOS and TRS5 SOS). In the derivative method, start of season (DER SOS) is defined as the date of the maximum in the first derivative. At the same time, the peak of the season (DER POS) corresponds to the maximum value of the curve (Tateishi and Ebata 2004). In the Gu method, the first derivative maximum was used to define the line tangent to the curve and thus their intersection with the baseline, describing the upturn date (GU UD), and with the peak line, representing the stabilization date (GU SD) (Gu et al. 2009). In the Zhang method, the onset (Greenup) and offset (Maturity) of photosynthetic activity are calculated using two locals maximum in the curvature change rate (Zhang et al. 2003). The method for phenometrics extraction is hosted on Zenodo for public use (Bregaglio et al. 2023). Phenometric data were extracted from the MODIS pixels including the 20 hazelnut orchards points. In addition, on all the MODIS pixels belonging to the 2019 Turkey hazelnut map, a spring phenology map based on the extracted phenometrics has been also derived.

**Table 1:** Descriptions of remote sensing phenometrics and their acronyms

Metric	Description
DER SOS	Start of season, the day of the fastest vegetation index increase (Tateishi and Ebata 2004).
DER POS	Peak of season, the day of the maximum value in the vegetation index time series (Tateishi and Ebata 2004).
TRS2 SOS	Threshold 20%, the day when the vegetation index time series reaches 20% of its annual amplitude.
TRS5 SOS	Threshold 50%, the day when the vegetation index time series reaches 50% of its annual amplitude.
GU UD	Upturn date around which canopy photosynthetic capacity often begins to increase sharply (Gu et al. 2009).
GU SD	Stabilization date when the peak canopy photosynthetic capacity occurs based on recovery line (Gu et al. 2009).
Greenup	The date of onset photosynthetic activity (Zhang et al. 2003).
Maturity	Date at which plant green leaf area is maximum (Zhang et al. 2003).

### Statistical analysis

The temporal dynamics of phenological ground observations and remotely sensed-derived phenometrics from the 20 hazelnut orchards have been analyzed by violin plots, considering their distributions (25th, 50th, and 75th percentiles). To evaluate the agreement between phenometric dates versus each BBCH phase, we used the root mean square deviation (RMSD), as measure of overall difference, and bias, as measure of the systematic difference between satellite and ground observations. Finally, Spearman coefficient correlations (r_s_) (*p* < 0.05) between field observations and remote sensing-based phenometrics were computed considering hazelnut spring vegetative and female reproductive phases.

## Results

### Temporal dynamics of phenometrics and comparison with ground observations

**Fig. 4:**
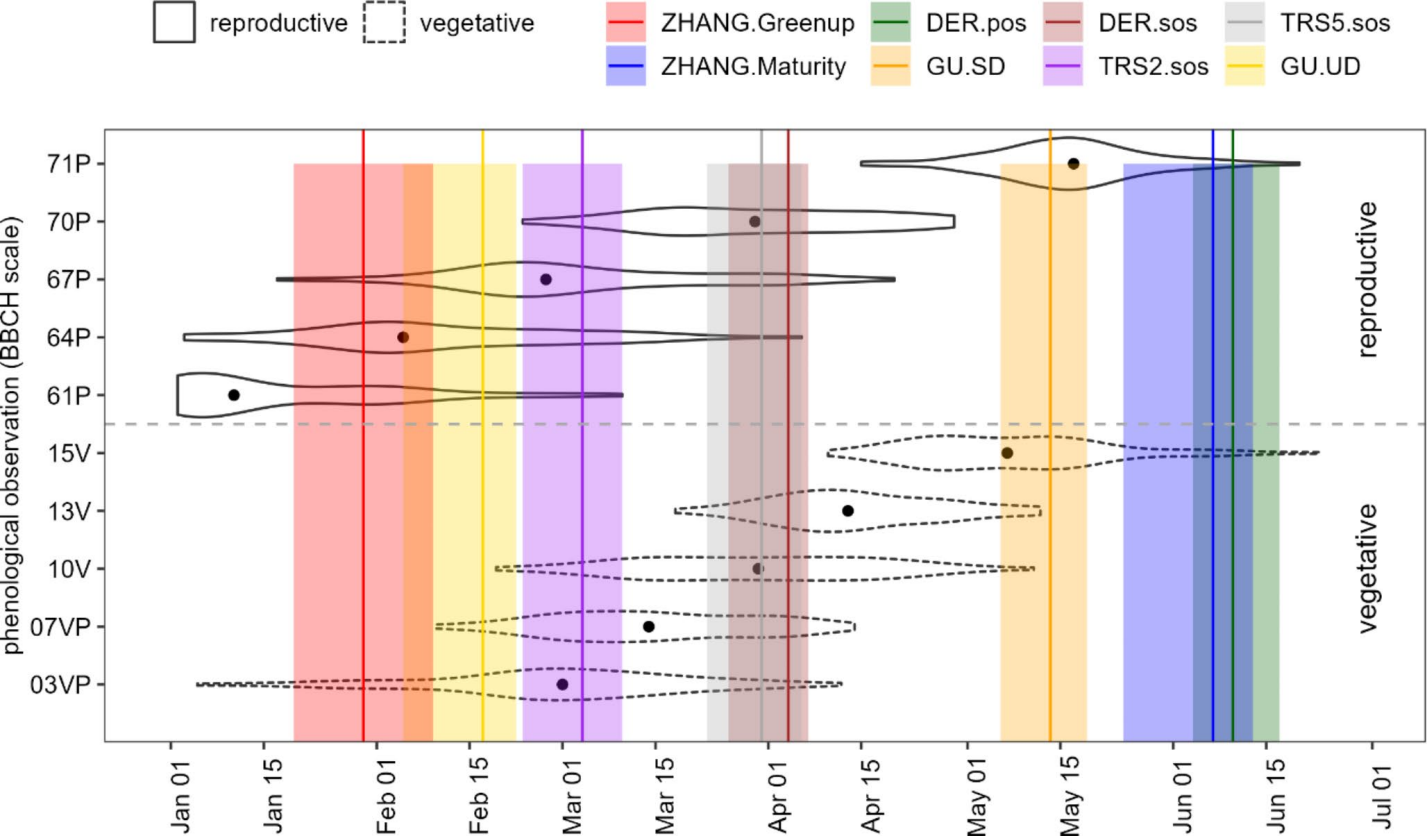
Temporal distribution of field observations of reproductive i.e. female flowering and fruit differentiation (solid violin plot) and vegetative (dotted violin plot) as compared to phenometrics from remote sensing (coloured line is the median, rectangles extend from 25th to 75th percentile) in the 20 hazelnut orchards in 2019–2022. Black dots represent the average values of phase times in the period of reference. See Fig. 3 for the BBCH phases description

The temporal distribution of ground and remote sensing-derived phenometrics in the 20 hazelnut orchards in 2019–2022 is shown in Fig. 4. The Greenup, GU UD, and TRS2 SOS metrics correspond to the early stages of the hazelnut spring phenology, being associated with the beginning of the greening onset; they have overlapped to BBCH 61P-67P (i.e., female flowering), and generally occurred before BBCH 03VP and 07VP phases (i.e., budbreak). On the contrary, GU SD, Maturity, and DER POS correspond to the phase of major canopy density and greenness peak, indicating the end of the spring vegetative dynamics, with GU SD occurring close to the BBCH 71P phase (i.e., clusters visible). The TRS5 SOS and DER SOS metrics represent the moments with the highest greening activity (more than 50% of EVI growth) and describe the steepest portion of the EVI curve, i.e., the greening and thickening process of the orchard canopy, i.e., between BBCH 70P (i.e., ovaries enlarge) – 07VP (i.e., budbreak) and 15 V (i.e., more leaves unfolded).

**Fig. 5:**
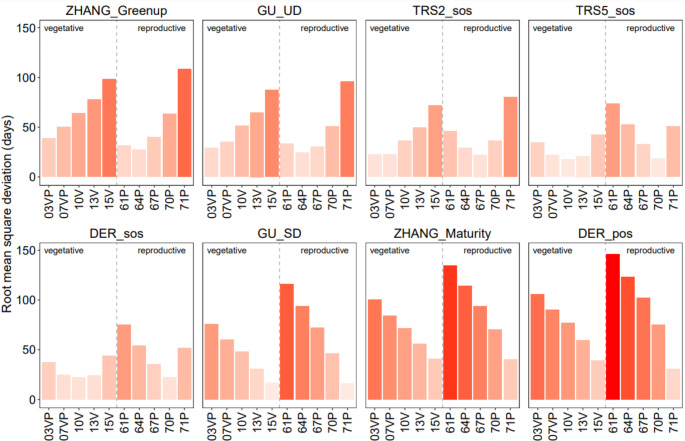
Root mean square deviation (RMSD) between phenological ground observations (BBCH code, x-axis) and remotely sensed phenometrics. Darker red indicates higher values of RMSD. See Fig. 3 for the BBCH phases description

**Fig. 6:**
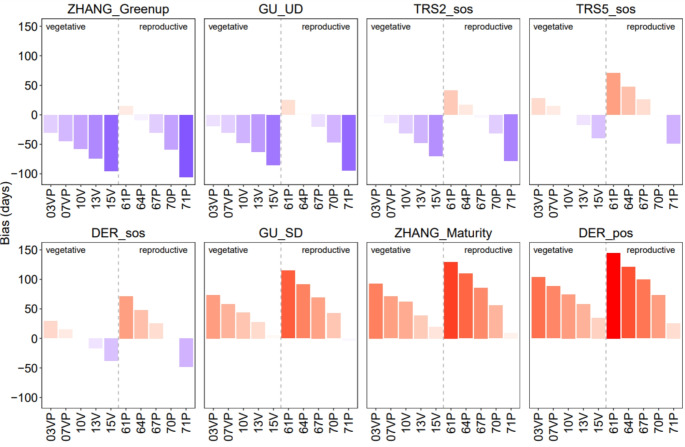
Bias between phenological ground observations (BBCH code, x-axis) and remotely sensed phenometrics. Positive values are highlighted in red, negative values in blue; darker color indicates higher levels of bias. See Fig. 3 for the BBCH phases description

According to Figs. 5 and 6, Greenup (median Jan 30) occurred on average about 15 days after BBCH 61P (bias = 15.22 days, RMSD = 31.37 days); GU UD (median Feb 17) occurred after BBCH 64P (bias = 2.1 days, RMSD = 24.35 days) and before 67P (bias=-20.46 days, RMSD = 30.43 days); TRS2 SOS almost overlapped with BBCH 03VP (bias=-3 days, RMSD = 22.77 days); TRS5 SOS (median Mar 30) and DER SOS (median Apr 3) occurred close to each other and to BBCH 10 V (bias=-0.46 days, RMSD = 17.90 and bias = 0.35 days, RMSD = 22.82 days respectively); GU SD (median May 12) occurred on average about 4 days before BBCH 71P (bias=-4.51 days, RMSD = 16.44 days); Maturity (median Jun 6) and DER POS (median Jun 9) were very close to each other and showed, on average, delays of more than 9 and 20 days from BBCH 71P, respectively (bias = 9.73 days, RMSD = 40.42 days and bias = 25.75, RMSD = 30.66 days respectively). This evidence means that (i) both flower blooming and ending occur prior to the 20% development of the vegetative process, (ii) TRS5 SOS and DER SOS are time-aligned with the emergence of leaves, and (iii) the cluster appearance is temporally close with the peak of the growing season.

**Fig. 7:**
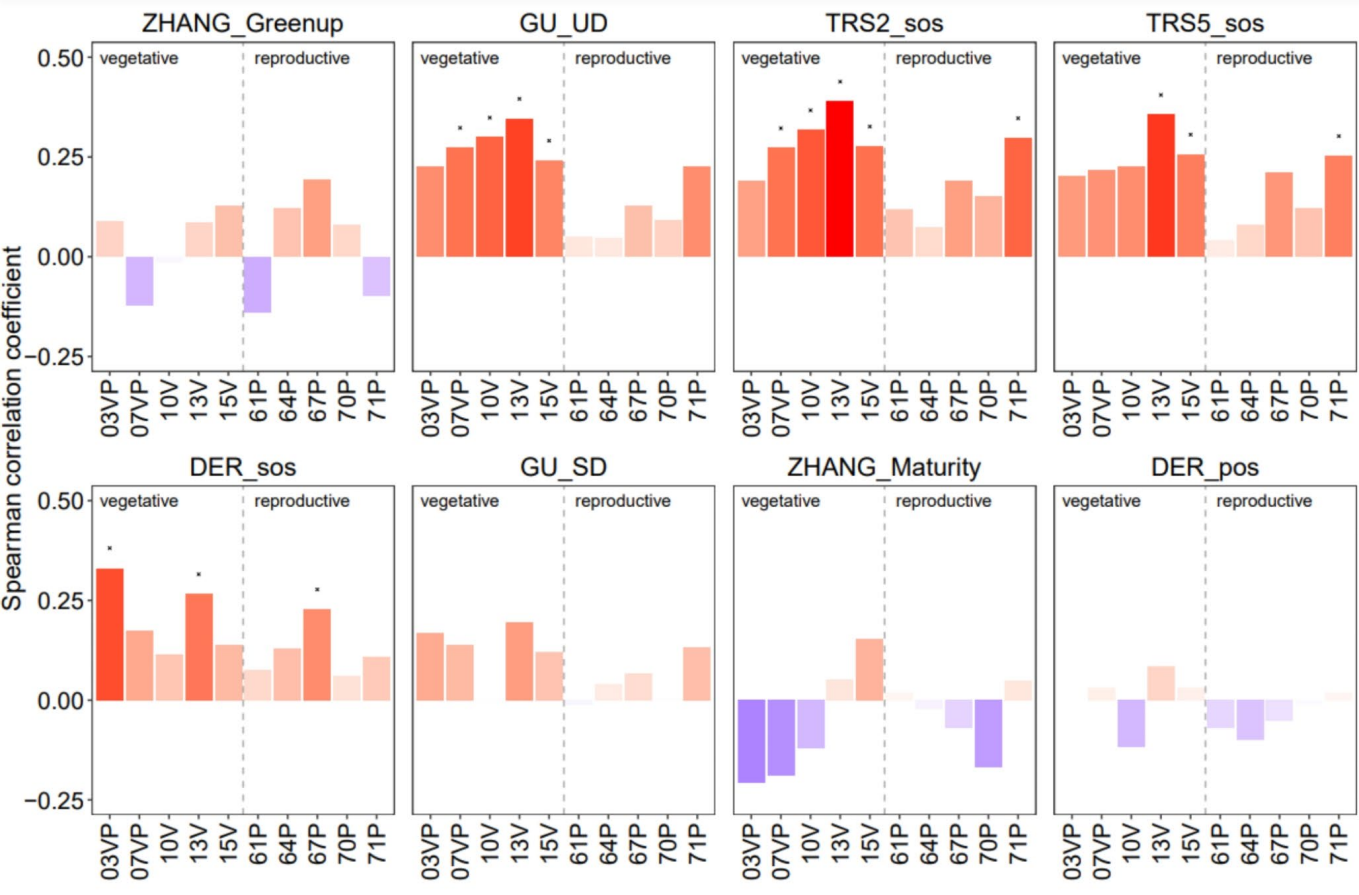
Spearman correlation coefficients between phenological ground observations (BBCH code, x-axis) and remotely sensed phenometrics. Positive correlations are highlighted in red, negative correlations in blue; darker color indicates higher correlation strength. The cross indicates significance at *p* = 0.05. See Fig. 3 for the BBCH phases description

The analysis of the correlation between hazelnut ground- and remotely sensed-observations highlighted that GU UD and TRS2 SOS were the phenometrics most positively correlated with vegetative BBCH phases (Fig. 7). The strength of the Spearman coefficient correlation (r_s_) increased from budbreak (BBCH 07VP; r_s_ = 0.27, *p* < 0.05), to leaf emergence (BBCH 10 V; r_s_ = 0.30 and 0.32 for GU UD and TRS2 SOS, respectively), and third leaf unfolded (BBCH 13 V; r_s_ = 0.35 and 0.39 for GU UD and TRS2 SOS, respectively). Significant (*p* < 0.05) but lower correlations emerged with the phase of more leaves unfolded (BBCH 15 V; r_s_ = 0.24 and 0.28 for GU UD and TRS2 SOS, respectively). The TRS2 SOS and TRS5 SOS metrics was also significantly correlated with reproductive phase of clusters visible (BBCH 71P; r_s_ = 0.32; r_s_ = 0.28, respectively). The metric DER SOS was positively correlated with early vegetative phases of swollen buds (BBCH 03VP; r_s_ = 0.33) and third leaf unfolded (BBCH 13 V; r_s_ = 0.27). The reproductive phenophase of end flowering (BBCH 67P) was positively correlated only with DER SOS metric (r_s_ = 0.23). The two ZHANG metrics Greenup and Maturity were weakly associated with all phenological phases, the latter showing negative correlations with most ground observations (BBCH 03VP, 07VP, 10 V, 67P, and 70P). Spearman coefficient correlation analysis was also performed distinguishing between pixels with less and more than 75% of hazelnut coverage and results are shown as Supplementary material S2. The two ZHANG metrics Greenup and Maturity were weakly associated with all phenological phases, the latter showing negative correlations with most ground observations (BBCH 03VP, 07VP, 10 V, 67P, and 70P). Spearman coefficient correlation analysis was also performed distinguishing between pixels with less and more than 75% of hazelnut coverage and results are shown as Supplementary material S2. When only pixels with a hazelnut coverage greater than 75% are considered, significant correlations emerge between BBCH 13 V (the third unfolded leaf), BBCH 15v (more leaves unfolded), and phenometric distributed across the entire phenological dynamics detected by satellite (Fig. S1). Conversely, when considering only pixels with hazelnut tree cover below 75%, many of the significant correlations between phenometrics and ground-detected phases are lost (Fig. S2).

For the correlated metrics, we observed that the GU UD metric occurred about 50 days before the BBCH 10 V phase (bias=-47.44 days, RMSD = 51.25 days), 65 days before the BBCH13V phase (bias=-63.16 days, RMSD = 64.89 days), 85 days before the BBCH15V phase (bias=-85.18 days, RMSD = 87.15), 30 days before the BBCH07V phase (bias=-29.91 days, RMSD = 35.10 days). DER SOS occurred about 30 days after BBCH03VP phase (bias = 29.38 days, RMSD = 37.63 days), 20 days before BBCH13V phase (bias=-16.56 days, RMSD = 24.60 days) and 25 days after BBCH67P phase (bias = 25.98 days, RMSD = 35.58 days). TRS2 SOS phase occurred about 15 days before BBCH07VP phase (bias=-14.10 days, RMSD = 22.59 days), 30 days before BBCH10V phase (bias=-31.20 days, RMSD = 36. 35 days), about 50 days before the BBCH13V phase (bias=-47.26 days, RMSD = 49.32 days), 70 days before the BBCH15V phase (bias=-69.73 days, RMSD = 71.79 days) and about 80 days before the BBCH71P phase (bias=-78.38 days, RMSD = 80.09 days). Finally, the TRS5 SOS phase occurred about 20 days before the BBCH13V phase (bias=-16.8 days, RMSD = 21.04 days), 40 days before the BBCH15V phase (bias=-39.29 days, RMSD = 42.32 days) and 50 days before the BBCH71P phase (bias=-48.32 days, RMSD = 50.64 days). These results confirm that the RMSD and bias values are similar for the phenometrics significantly correlated with the BBCH phases (Figs. 5 and 6). This confirms that the relationships between RS phenometrics and BBCH phases are consistent and stable, with interannual variations occurring in a directional manner.

## Discussion

Notwithstanding the inevitable disparities between ground-based observations and satellite-derived phenometrics at both temporal and spatial scales, several studies have demonstrated that satellite data can reliably estimate phenology across different environments and vegetation types (Fisher and Mustard 2007; Bajocco et al. 2019a). However, satellite phenological metrics rely on pixel greenness, encompassing heterogeneous plant communities, while ground observations are based on morphological traits in individual plants. This mismatch leads to a lag between the phenophases detected by remote sensing and field data (Wang et al. 2017; An et al. 2021).

In this study, the landscape examined was predominantly characterized by the presence of hazelnut orchards, although given the MODIS coarse spatial resolution (250 × 250 m), pixels showing a mixed coverage were present. Each pixels encompassed different percentages of hazelnut coverage for each reference orchard, as well as different types of surrounding environments, like crop fields, roads and settlements (Fig. 3). This spatial heterogeneity and the associated phenological variability within coarse-scale MODIS pixels lead to a reduction in the accuracy of satellite-derived hazelnut spring phenology. This contributed to the different levels of matching between ground- and satellite-based phenological observations, aligning with previous research (Fisher and Mustard 2007; Richardson et al. 2018; Bórnez et al. 2020; Ferrara et al. 2023). The observed discrepancy revealed that remotely sensed hazelnut spring phenometrics occurred with varying time lags from the corresponding field observed BBCH phases (Fig. 5). Metrics characterized by longer time lags (e.g., GU UD and Greenup) were associated with phenophases occurring in the initial segment of the EVI curve, i.e., between the initial steep growth rate and 20% of the total seasonal amplitude. During this time frame, full bloom and end flowering phases are detected on the ground. As a result, such metrics detected the very onset of the vegetation season, which did not correspond to actual hazelnut canopy greening but rather indicated the greening of the understory layers. In contrast, the derivative metrics and the 50% of the seasonal EVI amplitude showed the closest alignment with hazelnut SOS as observed from the field surveys, despite the spatial mismatch. The discrepancy diminished as the vegetation progressed towards maturity, and full-canopy stages, resulting in a gradual homogenization of pixel and plot surface characteristics. The results also unveiled a distinct temporal gradient among the satellite-based phenometrics, indicating that they correspond to specific points of change in the increasing part of the EVI curve and, consequently, reflect different stages of spring vegetative development. This suggests their classification into three phases: those describing the early portion (Greenup, GU UD, and TRS2 SOS), which gradually detected the onset of the greening trend; those indicating the middle season dynamics (TRS5 SOS and DER SOS) which detect the periods of highest EVI growth rate; those describing the late portion (GU SD, Maturity, and DER POS) of the spring part of the growing season, which detect the periods of highest EVI growth rate. The early metrics displayed the highest variability in starting dates, primarily because they encompassed the earliest growth stages of different plant community layers, representing the vertical heterogeneity of the canopy structure (Donnelly and Yu 2021). As the growing season progresses and the canopy closes, the phenological response becomes more homogeneous, reducing the variability in SOS dates (Ferrara et al. 2023).

Notwithstanding the temporal mismatch, this study showed a significant correlation between EVI-based phenometrics and key reproductive phases; in detail, between TRS2 SOS and TRS5 SOS and 71P (i.e., clusters visible), and between DER SOS and 67P (i.e., end of flowering) (Fig. 7). On one side, the growing amount of greenness and foliar production has a significant correspondence with the clusters’ appearance, while the timing of the end of flowering has a direct relationship with the EVI profile inflection point. While the satellite-based detection of temporal changes in vegetative phenology is now established and widely accepted (Bajocco et al. 2019a), the satellite monitoring of reproductive phenology (e.g., flowering, fruiting) is more challenging to demonstrate and has received far less attention. However, some previous studies highlighted the potential of coarse resolution satellite data for predicting flowering occurrence by using several vegetation indices. For instance, Karlsen et al. (2009) found a significant correspondence between the MODIS NDVI-defined onset and the date when the annual accumulated pollen sum reaches 2.5% of the annual total in birch, in Norway; Zang et al. (2020) tested the potential of MODIS NDVI and Normalized Difference Yellowness Index (NDYI) in mapping and tracing canola flowering in China; finally, Bogawski et al. (2019) modelled the onset of *Betula pendula* flowering in Poland using MODIS thermal data. In line with these studies, our results demonstrated that, although coarse resolution remotely sensed observations could not directly detect flowering and fruiting, the dynamics of specific phenometrics across the growing season may provide indirect information about the temporal occurrence of such events.

**Fig. 8:**
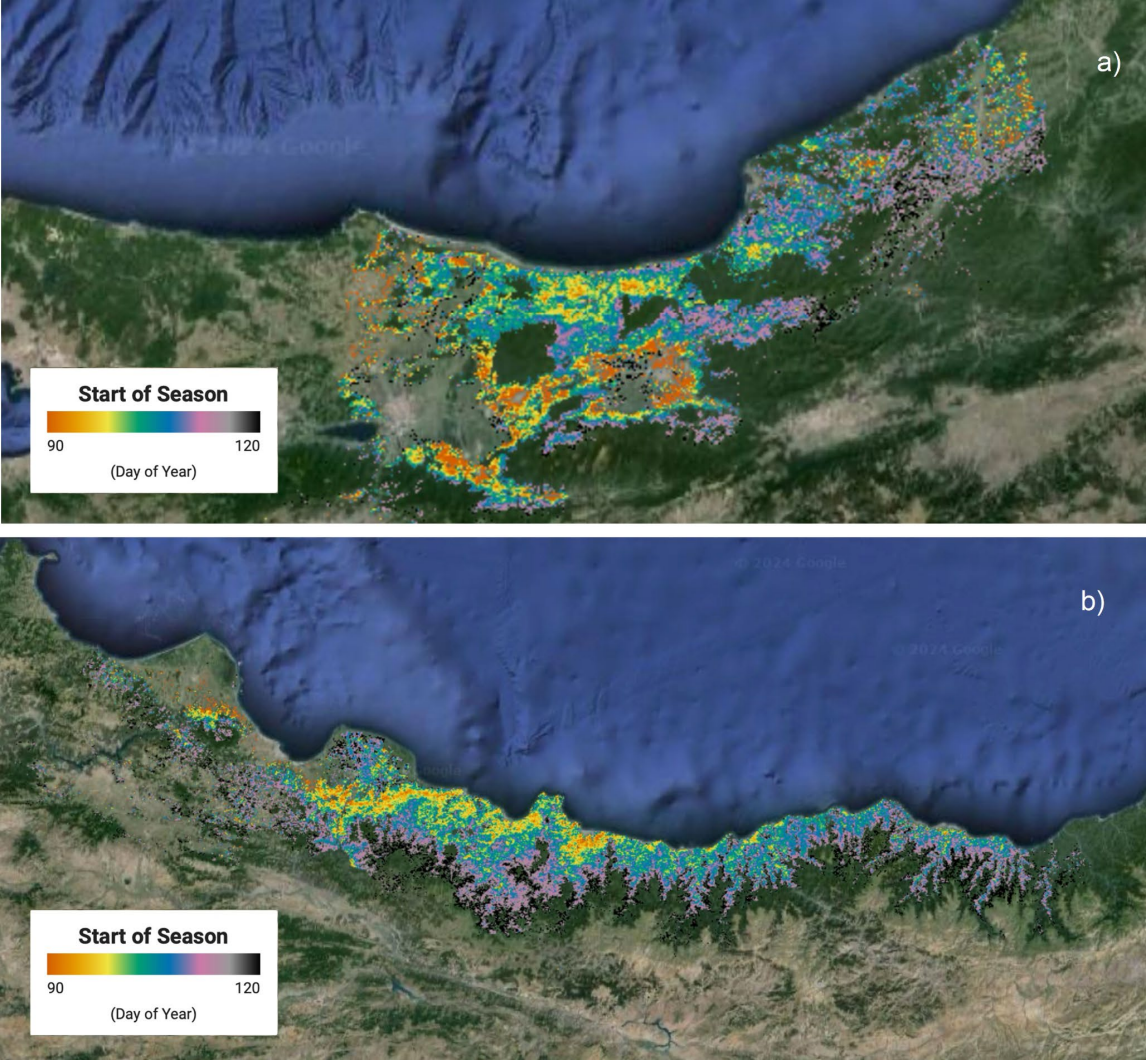
Example of spring phenology map of the hazelnut orchards distributed across Western (**a**) and Eastern (**b**) Black Sea region, according to the TRS5 SOS phenometric

The potential for transferring the findings from this study to operational in-season monitoring is demonstrated in Fig. 8, which presents a spring phenology map for the Turkish hazelnut-growing region, using TRS5 SOS data from 2019 as an example. The map illustrates the distribution of occurrence dates for TRS5 SOS, ranging from March 31 (day of year 90) to April 30 (day of year 120), highlighting distinct phenological patterns between the Western and Eastern Black Sea regions. In the Western region (depicted in Fig. 8a), the phenological distribution was scattered, with an early SOS observed in the western part (Sakarya, Duzce) and a late SOS in the eastern part (Zonguldak). Conversely, in the Eastern region (Fig. 8b) a clear spatial gradient of SOS was evident, shifting from later SOS in the inner mountainous areas to earlier SOS in the coastal zones (Samsun, Ordu, Giresun, Trabzon). These findings align with the relationship between TRS5 SOS and BBCH 13 V, 15 V, and 71P, suggesting that similar trends can be expected for these phenological phases. Moreover, based on the correlation analysis, as well as on RMSD and bias values, the systematic temporal difference between satellite- and ground-based observations may allow us to estimate the timing of these correlated phases and represent them in a spatially explicit manner.

In this framework, the findings of this study highlight the conceptual difference between the actual meanings of field-observed phenophases and remotely sensed phenometrics (Ferrara et al. 2023). In situ and remotely sensed phenological phases should be considered as two distinct types of events, potentially with distinct driving factors. Ground observations play a crucial role in studying plant life cycles and enhancing the accuracy of spring phenology forecasting models. On the other hand, despite having a coarser spatial resolution compared to traditional field approaches, satellite imagery makes phenological information spatially explicit and provides consistent data over time. In this context, ground and remote sensing data become essential for integrating the necessary information to study the dynamics of phenology events and establishing a reference base (Ferrara et al. 2023).

Understanding how satellite metrics align with ground observations can allow for the mapping of the spatial distribution of key phenological phases effectively; at the same time, the possibility to rely on easy-to-derive phenology map may facilitate the comprehension of spatial vegetation dynamics related to the growing season process and its drivers. This understanding represents a crucial step in improving data information accuracy, combining the strengths of multisource (satellite and field) data and significantly enhancing our ability to monitor the biotic response to climate change, extreme events, and drought stress (Studer et al. 2007).

The implications of our findings are significant for improving the interpretation of remotely sensed hazelnut phenology, which remains largely unexplored. Additionally, phenology is valuable in ecology for assessing the impact of extreme events, species interactions, and population migration while also benefiting human health by predicting pollen peaks for allergy sufferers (Koch et al. 2007; Meier et al. 2009). These are preliminary steps for coupling phenological data with coarse-resolution climatic variables and extending individual species’ phenological events to communities, which has been pursued for other plant species (White et al. 2005; Bajocco et al. 2019b).

## Conclusions

In this study, the extraction of phenological phases from satellite remote sensing data for hazelnut is carried out for the first time. A comprehensive ground observation dataset was leveraged to establish correlations with phenological metrics derived from MODIS EVI.

The release of remotely sensed phenological observations bolsters the scientific community’s resources for a deeper understanding of hazelnut vegetative development. These datasets are readily adaptable for a wide range of applications, including the calibration of crop models for enhanced yield forecasting. The open-sourcing of our data analytics workflow for extracting phenological information from remote sensing data empowers researchers and external parties to access hazelnut phenological data across regions in near-real time, thanks to the accessibility of MODIS data. Looking ahead, this research paves the way for the implementation of chilling and forcing phenological models, enabling a more precise characterization of thermal time intervals between phenological phases. This integration of findings promises to bridge the gap between the simulation of physiological processes and the observed hazelnut systems, both on the ground and from satellite observations, thereby advancing our understanding and predictive capabilities in hazelnut cultivation.

## Electronic supplementary material

Below is the link to the electronic supplementary material.

Supplementary Material 1

Supplementary Material 2

## Data Availability

We have not included authorization for the release of data, as the data supporting the findings of this study are not publicly available due to sensitivity concerns. Access may be granted to third parties on a case-by-case basis upon reasonable request.
