# Supplementary material for: Exploring the relationships between ground observations and remotely sensed hazelnut spring phenology
Source: Int J Biometeorol. 2024 Nov 8;69(2):281–94. doi: 10.1007/s00484-024-02815-1 (PMC11785673; doi:10.1007/s00484-024-02815-1)
Supplement: Supplementary file 1 — Supplementary Material 1 [file 484_2024_2815_MOESM1_ESM.pdf]

Weekly Monitoring

personal data

Phenological data

The 10 labelled Ocak must be checked and the prevalent phenological phase must be chosen

Vegetative development

Please mark only one of the option below

*

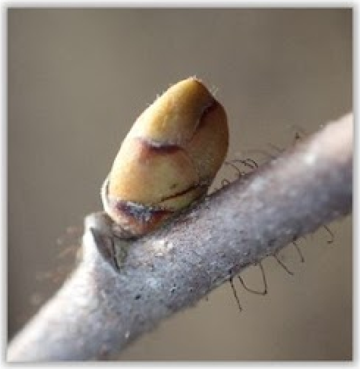

- [ ] V01_Dormant bud: Buds closed and covered by green, brown green or reddish scales according to cultivar

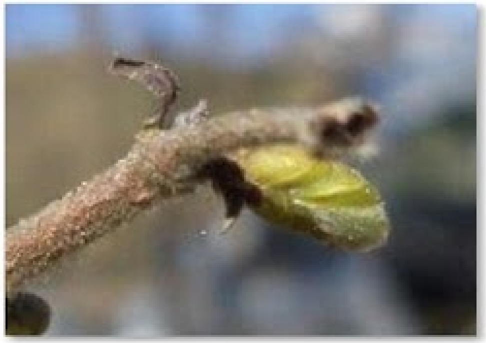

- [ ] V02_Swollen bud: Buds are visibly swollen and scales separated, this phase can be considered dormancy break

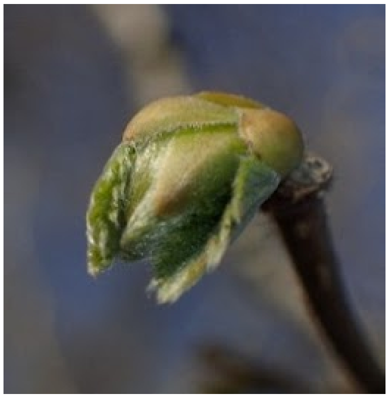

- [ ] V03_Budbreak: Buds opening. Green shoot tips just visible

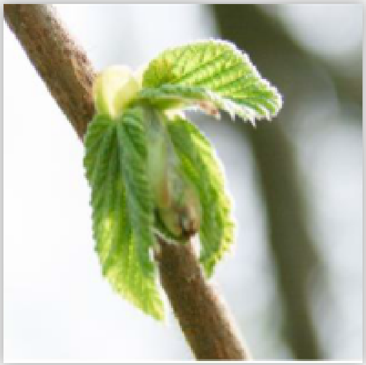

- [ ] V04_Leaf emergence: In at least 3 locations on the tree, first unfolded leaves are visible. A leaf is considered "unfolded" when the petiole or leaf stalk is visible

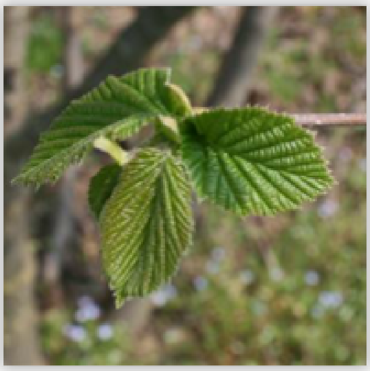

☐ V05_3rd leaf unfolded: > 50% of leaves are unfolded. Leaves are more separated without reaching their final size

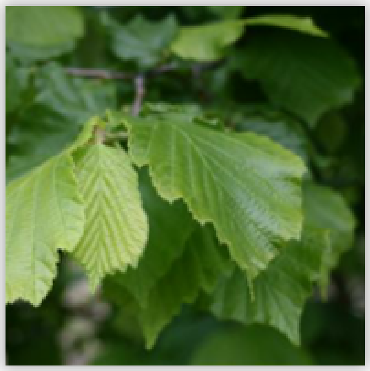

☐ V06_First mature leaves spotted: Presence of both young and mature leaves. The majority of leaves are unfolded

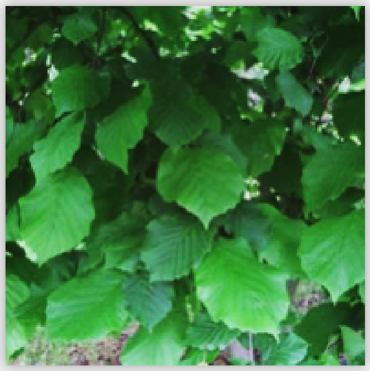

☐ V07_Mature leaves: For the whole plant, the majority (>90%) of leaves have elongated to their mature size

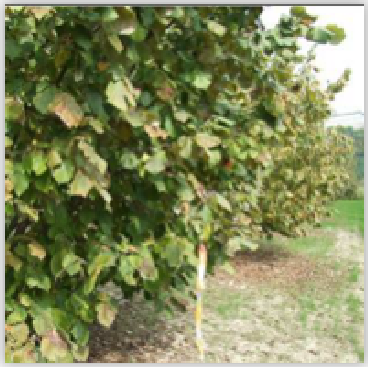

☐ V08_Leaf senescence: For the whole plant, > 50% of the leaves (including any that have fallen to the ground) have changed to their late-season colors

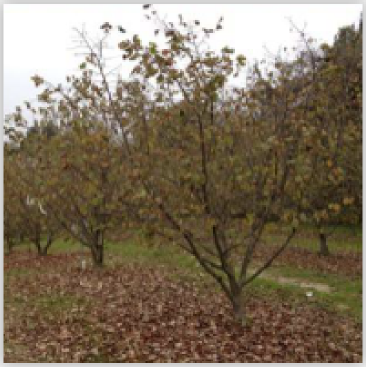

☐ V09_Leaf shedding: For the whole plant, > 50% of the leaves have

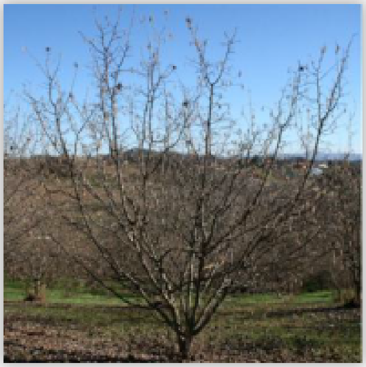

☐ V10_Bare tree: For the whole plant, virtually all (>90%) of the leaves

*

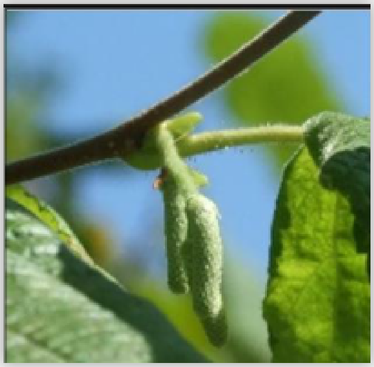

☐ R01_Catkins emergence: Catkins are visible but still developing

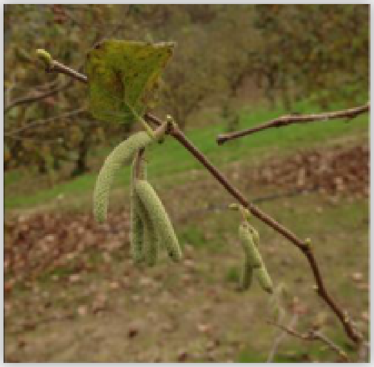

☐ R02_Catkins fully developed but still dormant: > 50% of the catkins are developed but still immature and completely closed, therefore pollen is not released yet

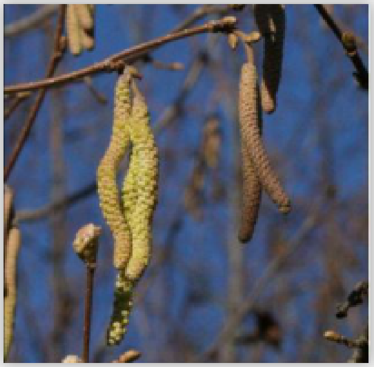

☐ R03_Some catkins begin to elongate: Catkins start to mature and turn yellow, pollen begins to be released. Immature catkins are still observed

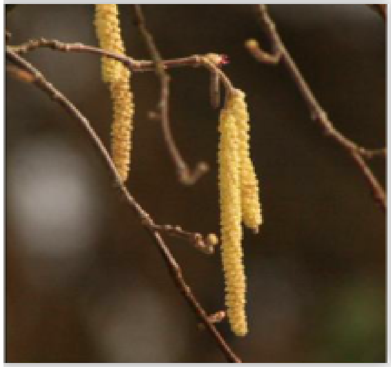

☐ R04_Catkins in full bloom: Most catkins have reached maturation, are fully elongated, anthers are completely open and pollen is released (anther dehiscence)

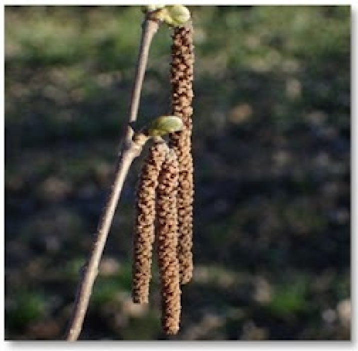

NA_If any of the previous phases applies

R05_Catkins withering: >50% catkins are withered and browned, do not bear pollen anymore, but they remain hanging on the branches

Female flower development
Please mark only one of the option below

*

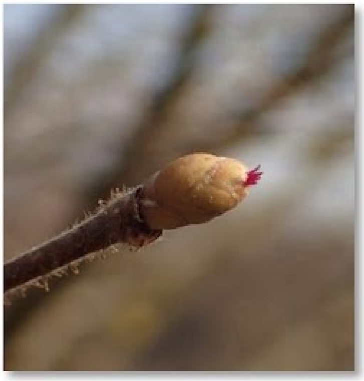

☐ R07_Beginning of female flowering: The red tips of the stigmas are visible and start to be receptive

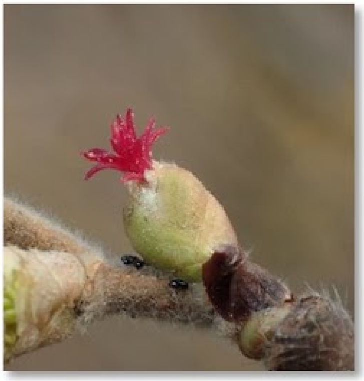

☐ R08_Inflorescence in full bloom: > 50% of the stigmas are fully extended. They become slightly sticky to the touch

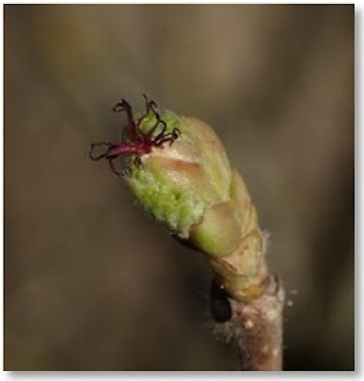

☐ R09_End of female flowering: > 50% of the stigmas are wilted and dark red (a sign that they have been pollinated)

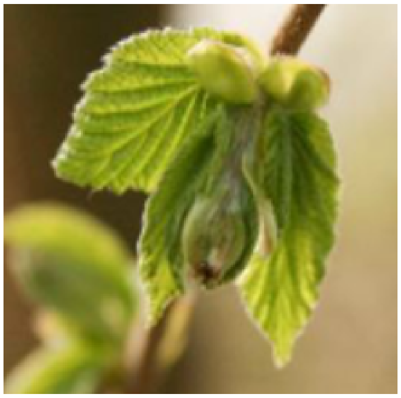

☐ R10_Ovaries enlarge: Ovaries are visible and they will grow slowly for some weeks until the resting sperm become activated and fertilization takes place

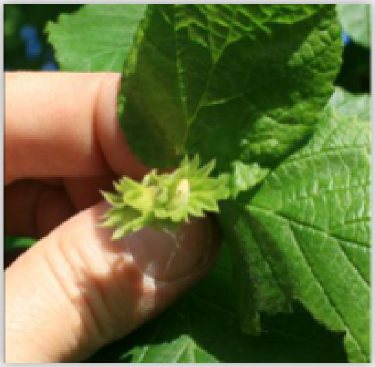

☐ R11_Clusters visible: Fertilization has occurred and the kernel will develop quickly. 50% of ovaries are now small fruits. The embryo is only visible at the microscope.

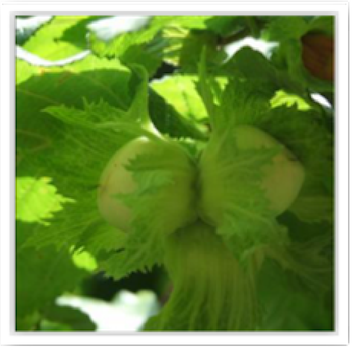

☐ R12_Inmature fruits: >50 % of fruits are developed but still immature and green. The embryo is visible with the naked eye.

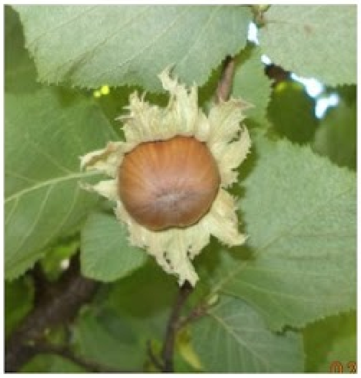

☐ R13_fruit maturation: > 50% of fruits reach maturity, turn brown and harden

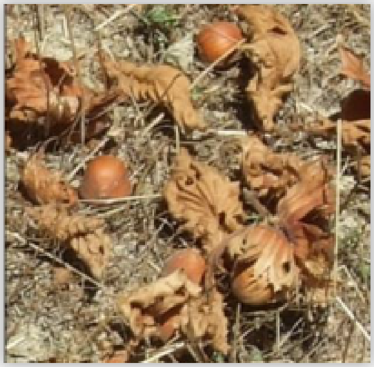

☐ R14_Fruit dropping: Beginning of the fruit dropping, but still many nuts are hanging in the tree

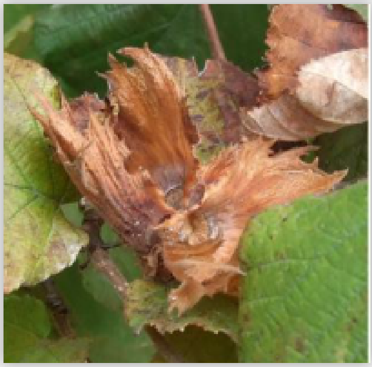

☐ R15_All nuts dropped: Harvest done

☐ NA_If any of the previous phases applies
