# Supplementary material for: Exploring the relationships between ground observations and remotely sensed hazelnut spring phenology
Source: Int J Biometeorol. 2024 Nov 8;69(2):281–94. doi: 10.1007/s00484-024-02815-1 (PMC11785673; doi:10.1007/s00484-024-02815-1)
Supplement: Supplementary file 2 — Supplementary Material 2 [file 484_2024_2815_MOESM2_ESM.docx]

**Supplementary Material S2**

**Figures**

**
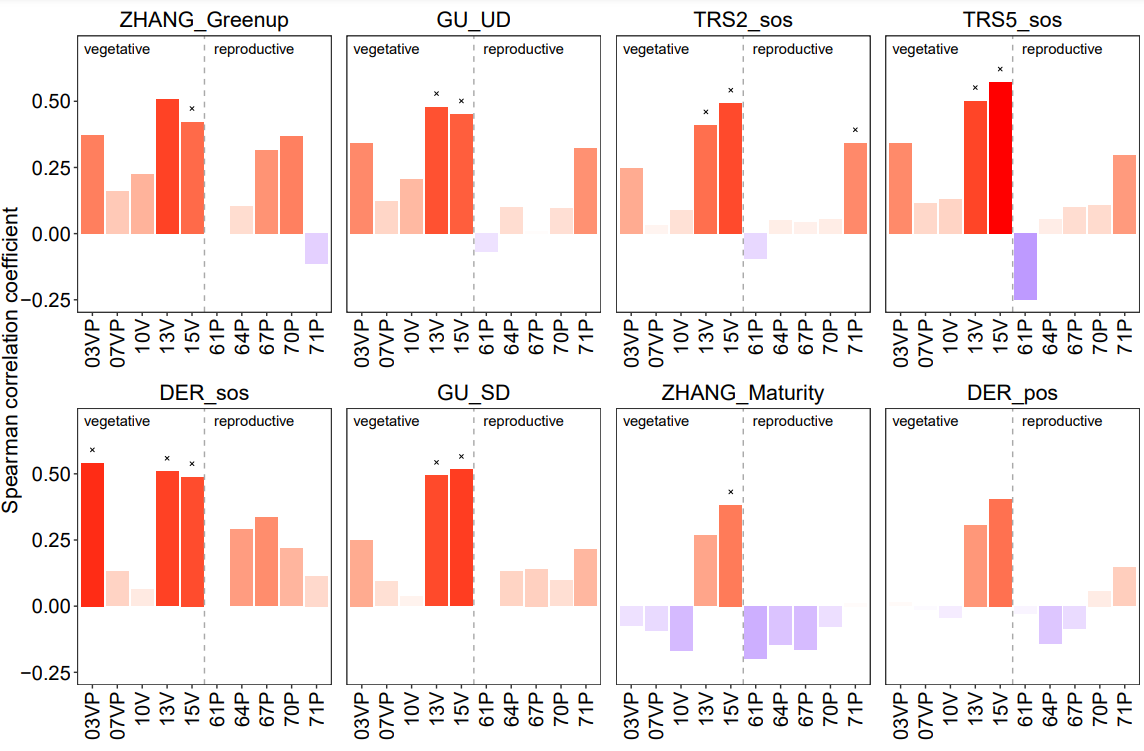
**

Fig. S1 Spearman coefficient correlation coefficients between phenological ground observations (BBCH code, x-axis) and remotely sensed phenometrics in pixels with more than 75% of hazelnut coverage (orchards ID02, ID06, ID10, ID11, ID14, ID16, ID18, ID19, ID20). Positive correlations are highlighted in blue, negative correlations in red with darker color indicate higher correlation strength. The cross indicates significance at *p* = 0.05.


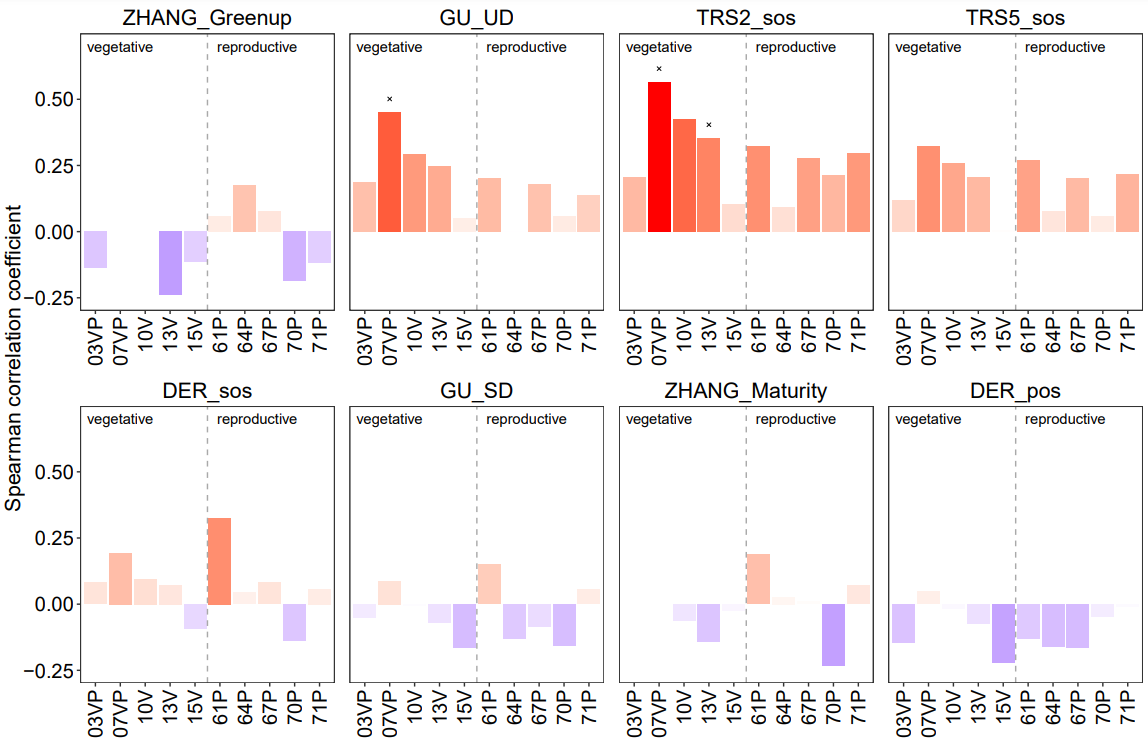


Fig. S2 Spearman coefficient correlation coefficients between phenological ground observations (BBCH code, x-axis) and remotely sensed phenometrics in pixels with less than 75% of hazelnut coverage (orchards (ID01, ID03, ID04, ID05, ID07, ID08, ID09, ID12, ID13, ID15, ID17). Positive correlations are highlighted in blue, negative correlations in red with darker color indicate higher correlation strength. The cross indicates significance at *p* = 0.05.
